# Beyond Psychotropic: Potential Repurposing of Fluoxetine toward Cancer Therapy

**DOI:** 10.3390/ijms25126314

**Published:** 2024-06-07

**Authors:** Sultan F. Kadasah, Abdulaziz M. S. Alqahtani, Abdullah Alkhammash, Mohamed O. Radwan

**Affiliations:** 1 Department of Biology, Faculty of Science, University of Bisha, P.O. Box 551, Bisha 61922, Saudi Arabia; 2 Department of Pharmacology, College of Pharmacy, Shaqra University, Shaqra 11961, Saudi Arabia; 3 Medicinal and Biological Chemistry Science Farm Joint Research Laboratory, Faculty of Life Sciences, Kumamoto University, Kumamoto 862-0973, Japan

**Keywords:** fluoxetine, cancer, repurposing, multidrug resistance (MDR)

## Abstract

Drug repurposing, rebranding an existing drug for a new therapeutic indication, is deemed a beneficial approach for a quick and cost-effective drug discovery process by skipping preclinical, Phase 1 trials and pharmacokinetic studies. Several psychotropic drugs, including selective serotonin reuptake inhibitors (SSRIs) and tricyclic antidepressants (TCAs), were studied for their potential application in different diseases, especially in cancer therapy. Fluoxetine (FLX) is one of the most prescribed psychotropic agents from the SSRIs class for the treatment of several neuropsychiatric disorders with a favorable safety profile. FLX exhibited different oncolytic effects via mechanisms distinct from its main serotonergic activity. Taking advantage of its ability to rapidly penetrate the blood–brain barrier, FLX could be particularly useful in brain tumors. This was proved by different in vitro and in vivo experiments using FLX as a monotherapy or combination with temozolomide (TMZ) or radiotherapy. In this review of the literature, we summarize the potential pleiotropic oncolytic roles of FLX against different cancers, highlighting the multifaceted activities of FLX and its ability to interrupt cancer proliferation via several molecular mechanisms and even surmount multidrug resistance (MDR). We elaborated on the successful synergistic combinations such as FXR/temozolomide and FXR/raloxifene for the treatment of glioblastoma and breast cancer, respectively. We showcased beneficial pharmaceutical trials to load FLX onto carriers to enhance its safety and efficacy on cancer cells. This is the first review article extensively summarizing all previous FLX repurposing studies for the management of cancer.

## 1. Introduction

Drug repurposing or repositioning represents an attractive approach for finding new applications for old drugs [1,2]. This kind of drug recycling introduces a batch of pros, including a shorter production time by eliminating Phase I clinical trials and a lower cost as a consequence in comparison to de novo drug discovery [1]. A pronounced merit of repurposing is that the pharmacokinetic and toxicity profiles of the repurposed entities were already identified. Previously, repurposing was dependent on serendipity; however, recently, it has been based on advanced omics technologies and computational tools [3]. One of the most outstanding examples is aspirin repurposing from a non-steroidal anti-inflammatory drug to an anti-platelet aggregation drug. Concomitantly, owing to the potential relationship between COX-2 and cancer, aspirin could be further reused for cancer therapy [4]. Drug repurposing modality played a role in trials to find drugs for COVID-19. Remdesivir and favipiravir are examples of existing drugs co-crystalized with SARS-CoV2 RNA-dependent RNA polymerase, whereas telaprevir and baicalein were co-crystalized SARS-CoV2 main protease (M^pro^) [5,6,7,8].

Cancer is still a major cause of death globally, accounting for one-sixth of global mortality [9,10,11]. Available drugs encounter resistance and sometimes possess intolerable undesirable effects [12]. The scientific community is usually urged to pursue alternative cancer chemotherapeutic agents to address the issues and resistance of existing drugs. One obstacle is the lengthy period of time required to develop one new drug, in addition to the huge cost and high possibility of failing clinical trials or facing pharmacokinetic issues [13].

**Figure 1:**
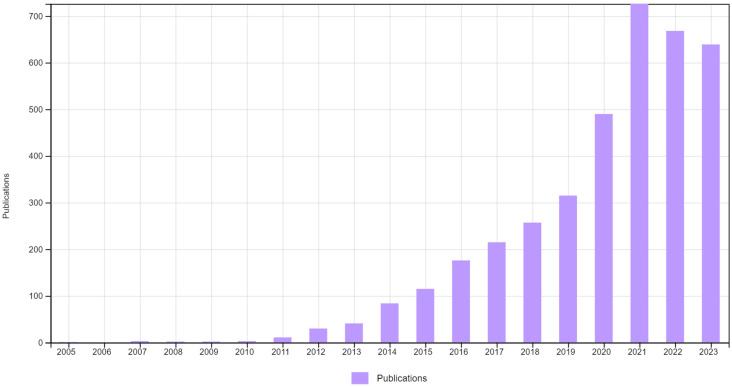
The number of scientific publications comprising scientific output linking repurposing studies with cancer over the period 2005–2023. Data were retrieved from the Web of Science database by using the keywords “repurposing” and “cancer”.

There are continuous trials demonstrating the repurposing of non-oncology drugs toward cancer therapy on both basic and clinical levels. Figure 1 demonstrates the remarkable increase in publications connecting cancer to repurposing, as found by a search in the Web of Science database. Sildenafil is a phosphodiesterase-5 inhibitor designed for the treatment of ischemic heart diseases and repurposed to treat erectile dysfunction. In addition, sildenafil is a sensitizer of cancer cells toward chemotherapy and radiation therapy [14,15]. Metformin, the widely used antidiabetic drug, exhibited anticancer and chemo-sensitization properties in preclinical and clinical studies [16,17].

The antimalarial drug quinacrine was found to be a dual target antiproliferative agent via the inhibition of Topo II and Hsp90 [18]. In addition, quinacrine was repurposed for managing cancer by several other mechanisms [19,20,21]. We have successful stories in the field of drug repurposing toward cancer management. Recently, we repositioned anti-HIV substituted benzimidazole derivatives for cell migration inhibition targeting heterogeneous nuclear ribonucleoprotein-M (hnRNP-M) [22]. Additionally, we repurposed *S*-trityl L-cysteine and *S*-trityl cysteamine derivatives from kinesin Eg5 inhibitors to Sirtuins 2 inhibitors [23,24]. A batch of repurposed non-oncology drugs for cancer management was extensively reviewed elsewhere [13,25].

Antidepressant drugs have a remarkable role in the therapy of cancer patients who are prone to depression disorders [26,27]. Early observations showed conflicting findings on the antidepressants’ effect on cancer promotion and growth [26,28]. Later studies revealed the great potential of antidepressant drugs, including tricyclic antidepressants (TCAs) and selective serotonin reuptake inhibitors (SSRIs), for repurposing to cancer therapy via several mechanisms of action [2,8,27,29,30]. Different psychotropic drugs, including fluoxetine, fluspirilene, ebastine, and aripiprazole, demonstrated anticancer activity in glioblastoma, colorectal carcinoma, and breast cancer cell lines by disrupting lysosomal and mitochondrial function and triggering cell death but not inducing apoptosis [31]. In fact, among all classes of antidepressants, SSRIs have the highest repurposing potential for the management of cancer [2,32].

Owing to their favorable safety profile, SSRIs are the most prescribed antidepressant drugs, and they are used as adjuvant therapy for the treatment of other neuropsychiatric disorders [33,34]. Basically, a three-year study on a larger sample of patients using SSRIs ruled out any breast cancer risk due to their administration [35]. Concomitantly, several SSRIs proved oncopreventive and/or oncolytic properties against cancers of the lung [36], colorectal [37,38], and breast [39]. Accumulating pieces of evidence confirmed that SSRIs’ oncolytic activity is mainly through independent actions of their primary serotonergic-mediated mechanisms [30].

Indeed, all SSRIs showed various oncolytic activities except for vilazodone [2]. For example, sertraline (Zoloft^®^) induces apoptosis in colon cancer cells [40], suppresses tumor growth by blocking the 5′ adenosine monophosphate-activated protein kinase/mammalian target of rapamycin (AMPK/mTOR) pathway, promotes autophagic flux in non-small cell lung cancer (NSCLC) cells [41], shows synergistic effects with sorafenib against hepatocellular carcinoma (HCC) cells proliferation [42], and reduces breast cell growth by interrupting serine/glycine synthesis [43,44].

Paroxetine (Paxil^®^) induces apoptosis in NSCLC via the ROS-MAPK pathway [45] in colon cancer cells by suppressing MET and HER3 kinases [46], and in MCF-7 by increasing extracellular calcium ion (Ca^2+^) and p38 [32]. Citalopram (CeleXA^®^) has a pro-apoptotic effect on acute myeloid leukemia (AML) via caspase-3 activation. Notably, it lowers invasion and metastasis of colon cancer cells by inhibiting the transforming growth factor-β (TGF-β) signaling pathway. The *S*-(+)-enantiomer of citalopram, escitalopram (Lexapro^®^), induces apoptosis and autophagy in glioblastoma [47] and NSCLC [48] and suppresses breast cancer cell growth [49].

**Figure 2:**
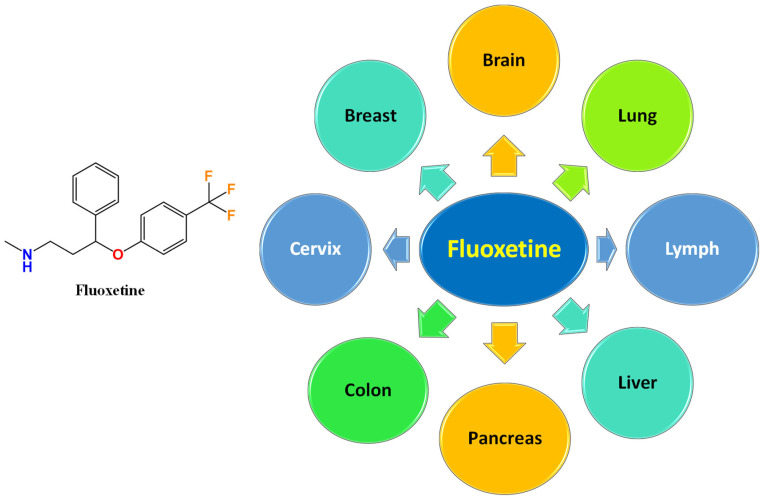
Chemical structure of fluoxetine (FLX) and summary of different sensitive cancers to fluoxetine treatment.

Fluoxetine (FLX; Prozac^®^; see Figure 1) was the first approved SSRI and is still one of the most prescribed antidepressants worldwide. The literature is full of interesting studies on the potential rebranding of FLX for the management of different cancer types. Among different antidepressants, only FLX improved the overall survival of patients receiving FLX/PD-1/L1 immunotherapy compared to only PD-1/L1, according to a cohort study on cancer patients who receive checkpoint inhibitors [50]. For the first time, we introduce a review article to emphasize the potential role of FLX in the management of neoplasms, either solely or in combination with other chemotherapeutic agents. Additionally, the role of FLX in overcoming multidrug resistance (MDR) is discussed. Figure 2 summarizes the different cancer types that can be modulated by FLX. This review further includes pharmaceutical trials to load FLX on carriers to control its cellular release and enhance its efficacy.

## 2. Materials and Methods

We explored the literature in PubMed, Google Scholar, and Web of Science databases using three keywords, “fluoxetine”, “repurposing”, and “cancer”, to conduct a comprehensive search. Our search was carried out without year limitations because there was no previous review article on this topic. The outcome was around 92 research and review articles, of which 51 were extensively considered for the current review article. The residual articles were not investigated thoroughly because they mainly focused on repurposing other antidepressants or antipsychotics. We divided the results into sections based on the target cancer type. To recognize the potential cancer-related targets of FLX, the Swiss Target Prediction tool (http://www.swisstargetprediction.ch/) was accessed on 10 March 2024 [51].

## 3. Results

### 3.1. FLX in Brain Tumors

For brain tumors like glioblastoma multiforme (GBM), the blood–brain barrier (BBB) may hinder the development of some drugs that efficiently stop GBM cell proliferation and trigger apoptosis in vitro [30,52]. In other words, the efficacy of GBM drugs is often faced by poor drug delivery due to the presence of the BBB [53]. As an antidepressant drug, FLX has favorable physicochemical and pharmacokinetic properties to bypass BBB; hence, its repurposing for GBM therapy is substantially useful [54]. De facto, previous medical records showed remarkably enhanced life expectancy in GBM patients taking fluoxetine, but not other SSRIs [55].

FLX exhibited in vitro apoptotic effects on human GBM cells, viz. U87 and GMB8401, by increasing the intracellular Ca^2+^ concentration, damaging mitochondrial membranes, and releasing apoptogenic factors at 25–30 μM. This effect was reversed by the coadministrations of (2,3-dioxo-6-nitro-7-sulfamoyl-benzo[f]quinoxaline) (NBQX), a blocker of α-amino-3-hydroxy-5-methyl-4-isoxazolepropionic acid receptor (AMPAR), which is highly expressed in GBM. In silico calculations supported by experimental tools emphasized that FLX binds to the GLUR1 subunit of AMPAR [56,57]. The apoptotic effect was consistent in vivo in a tumor xenograft using U87 GBM cells, using 10 mg/kg of FLX. Of note, the apoptotic effect was significant in brain tumors but not in normal brain cells of mice, reflecting a favorable safety profile of FLX [53].

Temozolomide (TMZ) is one of the most effective chemotherapeutics for GBM. Due to its common resistance, Wang’s group studied FLX activity in GBM and its synergistic effect with TMZ. Only FLX was able to inhibit the growth of different rat and human GBM cells, including C6, U87-MG, U373, and U251 with respective IC_50_ 14.7, 21.8, 48.5, and 22.9 μM. FLX clearly enhanced C6 apoptosis by increasing the caspase-3 concentration and endoplasmic reticulum stress (ERS). Mechanistically, FXL elevated the expression of C/EBP homologous protein (CHOP) and autophosphorylation of its upstream and downstream signaling pathways, as identified by Western blotting. Notably, FLX sensitized C6 cells to TMZ treatment, showing a synergistic antiproliferative effect at lower inhibitory concentrations. The latter effect is also mediated by the CHOP pathway, as the knockdown of CHOP omitted FXR/TMZ synergism [58].

Considering that the TMZ resistance is mainly caused by DNA-repair O6-methylguanine-DNA methyltransferase (MGMT), Song et al. studied the role of FLX in suppressing MGMT expression. The authors concluded that 10 μM of FLX significantly reduced MGMT concentrations by disrupting NF-kB/p65 signaling and, hence, sensitizing GBM cells to TMZ. Those in vitro results were further validated in vivo in a murine subcutaneous xenograft model of human GBM cells U-138 MG [59].

An elegant research work by Bi et al. in 2021 extensively showed the effect of FLX on the signaling and metabolism of GBM. They identified acid sphingomyelinase (ASM; sphingomyelin phosphodiesterase 1 (SMPD1)) as an attractive GBM target that is necessary for cancer cell survival. In GBM cells, fluoxetine inhibited the SMPD1 enzymatic-activity formation of ceramide from sphingomyelin, resulting in a dose-dependent death in three different types of GBM cells. Consistently, this effect was reproduced in orthotopic xenografts implanted in nude mice brains. Interestingly, this effect is accompanied by lysosomal stress and suppression of the overexpressed oncogenic epidermal growth factor receptor VIII (EGFRvIII), a constitutively active form mutation of EGFR [55]. The authors confirmed in vitro and in vivo FLX/TMZ synergism in inducing DNA damage and cell death in multiple GBM models. This is in accordance with the recorded higher survival rates of GBM patients who co-administer FLX/TMZ [55].

Owing to its favorable safety profile, a research group studied the possibility of FLX repurposing for fatal neuroblastoma in children, which accounts for 7% of cancer diagnoses of children under 15 years old. In neuroblastoma, the Myc oncoprotein amplification is used as a negative prognostic marker and is a hallmark of a high-risk disease. Myc regulates the CKS1/SKP2/p27^kip1^ axis, which can be interrupted by FLX. The latter inhibited CKS1, increased p27^Kip1^ expression, and triggered neuroblastoma cell death at 10 μM concentration. Furthermore, small doses of FLX reduced the invasiveness and metastasis of neuroblastoma cells. This effect was further validated in a xenograft mice model [60].

FLX is not only effective as a monotherapy or combination therapy with TMZ, but it could also sensitize GBM cells (U-87 MG) to radiotherapy. Surprisingly, FLX showed a radioprotective effect on normal fibroblast cells HFFF2 in vitro [61]. Conclusively, FLX (10 μM) represents the best SSRI in terms of brain cancer management. Its effect is remarkable when combined with TMZ or radiotherapy.

### 3.2. FLX in Breast Cancer

Breast cancer is the most spreading cancer among women, with potential fatal metastasis to the bones, liver, lungs, and brain [62,63]. The literature is loaded with considerable results showing the sensitivity of different types of breast cancer to FLX. The group of Chan took advantage of FLX to circumvent MDR in the MCF-7/ADM (doxorubicin-resistant human breast carcinoma) cells; they used stealth liposome co-encapsulation of doxorubicin and FLX for prolonged circulation half-life and improved safety in vivo. The used formulation demonstrated promising anticancer properties, under both in vitro and in vivo conditions, capable of the effective reversal of doxorubicin resistance [64].

The mechanism of FLX for reversing MDR was not fully understood. Thus, another group studied the synergism of FLX with Adriamycin (ADM) and paclitaxel (PXL). FLX-ADM combination—(5 μg/mL) FLX with various concentrations of ADM—enhanced apoptosis significantly in MCF-7/ADM-resistant cells but not MCF-7 cells. The IC_50_ values of sole ADM and ADM with FLX were 13.62 µM and 2.71 µM, respectively, implying a significant positive effect of FLX addition. The authors found that the chemo-sensitizing effect of FLX occurs via simultaneous upregulation of the tumor suppressor protein p53 and downregulation of B-cell lymphoma 2 (Bcl-2) [65].

Raloxifene (RAL) is widely used for the treatment of estrogen receptor-positive breast cancer, with fewer side effects than the parent tamoxifen [66,67]. Kabel et al. investigated the potential synergistic effect in the case of using a FLX/RAL combination in experimentally 7,12-Dimethyl Benzanthracene (DMBA)-induced breast cancer in female Wistar rats. They found that either FLX or RAL is an effective pro-apoptotic agent, but the RAL/FLX combination had a better outcome than either of them, as shown by the tumor volume size. RAL (3 mg/kg)/FLX (30 mg/kg) administration improved tumor antioxidant status via the elevation of catalase (CAT) and superoxide dismutase (SOD) and reduction of malondialdehyde (MDA) in tumor tissue compared to the control group. The titled combination reduced tissue concentration of the pro-inflammatory cytokines such as tumor necrosis factor-α (TNF-α) and interleukin-6 (IL-6) and suppressed TGF-β [68].

A complementary study to the abovementioned one regarding the FLX/RAL combination showed that it can suppress invasion, metastasis, and angiogenesis of DMBA-induced breast cancer. The combination lowered mammary tissue vascular endothelial growth factor (VEGF), macrophage colony-stimulating factor (M-CSF), and matrix metalloproteinase-9 (MMP-9) levels, as determined by an enzyme-linked immunosorbent assay (ELISA). In accordance with the previous study, the FLX/RAL oncolytic effect significantly surpasses each sole drug [69].

According to Duarte et al., the IC_50_ of FLX against MCF-7 cells is 7.78 µM, as detected by MTT (3-(4,5-Dimethylthiazol-2-yl)-2,5-Diphenyltetrazolium Bromide) assay. When combined with PXL, a significant synergistic antiproliferative effect was observed using both MTT and SRB assays [70]. The same research group found significant synergism for the combination of FXL and doxorubicin against MCF-7 in another study [71]. They also found that cotreatment of FXL and honeybee venom demonstrated a better cytotoxic effect than either of them [72].

In a study testing different antidepressants, including amitriptyline, bupropion, FLX, paroxetine, and tianeptine, as monotherapy for the induction of apoptotic cell death in breast cancer cells MCF-7, only paroxetine, followed by FLX (10 µM), demonstrated significant activity in a dose-dependent manner [32]. Another study benchmarked standalone FLX on triple-negative breast cancer (TNBC) in vivo. Surprisingly, FLX suppressed tumor growth by downregulating STAT3 signaling transduction and triggering a caspase-mediated apoptotic pathway [73].

By virtue of the aggressiveness of TNBC, Bowie et al. extensively studied the effect of FLX on TNBC cells SUM149PT. They found that treatment with 10 µM FLX induced apoptosis and enhanced ER stress and autophagy (IC_50_ µM 7.9). This is accompanied by cell-cycle arrest at the G1 phase and caspase-7-mediated cell death. Those effects are less prominent in the non-transformed MCF-10A cells, reflecting a favorable safety profile [74].

Another research group studied the FLX effect on other TNBC cells, MDA-MB-231 and MDA-MB-436. Sun et al. revealed that 1 μmol/L FLX induced both apoptosis and autophagic cell death in the titled cell lines. The apoptotic effect is attributed to the upregulation of the expression levels of caspase-3 and caspase-8 and poly (ADP-ribose) polymerase (PARP). In addition, FLX significantly reduced the phosphorylation of eukaryotic elongation factor-2 kinase (eEF2K), which is overexpressed in different cancers and plays an indispensable role in the crosstalk between apoptosis and autophagy in TNBC. Furthermore, FLX modulates autophagic proteins; it decreases phosphorylation of mTOR, activates AMPK, and increases ULK1 phosphorylation [75].

### 3.3. FLX in Hepatocellular Carcinoma (HCC)

Hepatocellular carcinoma (HCC) is the most common form of primary liver malignancy, with 906,000 new cases and 830,000 deaths worldwide yearly, making it the third leading cause of cancer death [76,77]. Several SSRIs and SNRIs, not including FLX, were tested for their possible cytotoxic effect on HCC [78]. FLX reduced the viability of the human HCC cell line Hep3B and induced apoptosis, as detected by MTT assay and staining, where at 10, 30, and 100 μM of FLX treatment for 24 h, the viability values were 98.3 ± 15.2, 80.4 ± 4.3, and 30.4 ± 3.4%, respectively. Disruption of mitochondrial membrane potential (MMP) is an early indicator of reactive nitrogen species-induced apoptosis. The authors found that the treatment of Hep3B with FLX resulted in a loss of MMP and enhanced the formation of reactive oxygen species (ROS). This was accompanied by the suppression of anti-apoptotic phosphorylated extracellular signal-regulated kinase 1/2 (pERK1/2) protein and an increase in pro-apoptotic c-JUN N-terminal kinase (c-JNK) and p38 mitogen-activated protein kinases (MAPKs) [79].

An in vivo study showed that FLX significantly suppressed tumor growth of Hep3B cells in a xenograft mice model without inducing liver pathology or general toxicity at a 10 mg/kg FLX dose. The oncolytic effect is attributed to the upregulation of the extrinsic and intrinsic apoptosis-associated proteins, including caspase-3, caspase-8, and caspase-9, and suppression of metastasis-associated protein VEGF, MMP-9, urokinase-type plasminogen activator (uPA), and Cyclin- D1, as validated by immunohistochemistry (IHC) staining. This indicated that FLX could reduce Hep3B angiogenesis and cell invasion. Additionally, FLX attenuated phosphorylation of nuclear factor-kappa B (NF-κB) p65 on ser276, AKT, and ERK in the titled cells as validated by IHC [80]. A similar action was induced by 30 µM FLX treatment of HCC SK-Hep1 cells for 48 h in another study for the same research group [81].

Hend et al. prepared FLX-loaded hexosome (HEX) using the hot emulsification method to prolong the release and enhance the activity of FXR on HCC cells HepG2. Successfully, the optimized HEX was able to prolong FLX release, where only 19.5% was released in PBS pH 7.4 after 24 h, which was highly increased in acidic medium pH 5.5, which imitates the cancer microenvironment. Accordingly, HEX improved/enhanced the cellular delivery and cytotoxic activity of FLX against HepG2 compared to the drug solution [82].

### 3.4. FLX in Colon Cancer

Colorectal cancer is ranked as the third most common cancer in the United States [83,84]. MTT assay showed a significant antiproliferative effect of FLX on colon cancer cells HT-29 with an IC_50_ value of 6.12 µM. However, the cotreatment of FLX and 5-fluorouracil (5-FU) (IC_50_ 3.79 µM) did not show a synergistic effect at different concentrations [70]. Nevertheless, the combination of fluoxetine and honeybee venom demonstrated a significant synergistic effect against HT-29 cells [72]. Another study showed that (20 µM) FLX remarkably augmented the antiproliferative effect of either cisplatin or carboplatin but not oxaliplatin on colon cancer cells HCT116. The authors attributed this synergistic effect to calmodulin inhibition by FLX [85].

Another effective combination of metformin/Efavirenz/FLX (5 mM/1.5 µM/0.9 µM) against HCT116 human colon cancer cells was recently reported by Kang et al. [86]. The three-drug combination triggered a massive increase in ROS levels and exhibited a dramatic antiproliferative effect on HCT116 but not human dermal fibroblasts (HDF) cells. The treated HCT116 cells showed increased DNA damage, apoptosis, autophagy, and necroptosis-related factors, as detected by Western blotting. In the HCT116 xenograft mice model, the titled combination showed consistent results by reducing tumor weight and size compared to the control group [86].

Another validating study showed that FLX-induced concentration-dependent apoptosis and DNA fragmentation in HCT116^+^/^+^ and p53 gene-depleted HCT116^−^/^−^ human colorectal cancer cells with respective IC_50_ values of 3.19 ± 0.23 μM and 4.73 ± 0.5 μM and less effect on normal cells. Mechanistically, FLX treatment resulted in cell-cycle arrest at Sub-G1 and G0/G1 phases in both cell lines, as detected by a FACS analysis [87]. In summary, the molecular mechanism of FLX cytotoxicity against HCT116 cells is independent of p53 modulation.

### 3.5. FLX in Cervical Cancer

Cervical cancer is one of the most spreading cancers in females worldwide [10]. The resistance of cervical cancer cells to cisplatin is a real challenge that leads to a decline in the survival rate [88]. In cervical-cancer HeLa cells, 50 μM of FLX reduced viability via energy depletion and increased cytosolic Ca^2+^ concentration by emptying the endoplasmic reticulum (ER) through the translocon, an ER Ca^2+^ leakage structure [89].

In a dose-dependent manner, FLX promoted HeLa cellular chemo-sensitivity to cisplatin in vitro and in xenograft mice models compared to FLX or cisplatin alone. The titled combination, 16 µM FLX/16 µM cisplatin, triggered G0/G1 phase arrest (73%) compared with FLX (58%) or cisplatin (60%), as observed by FACS analysis. Additionally, the percentage of apoptotic cells was 48.3% compared with FLX (38.1%) or cisplatin (31.5%). In vivo assay showed that oral dose of FXR (1 mg/kg), cisplatin (3 mg/kg), and FXR/cisplatin combination effectively reduced the tumor weight with inhibition rates of 10.9%, 46.6%, and 53.7%, respectively. Mechanistically, FXR/cisplatin upregulated apoptotic proteins caspase-9 and p17 and suppressed MDR proteins glutathione S-transferase π (GSTπ) and P-gp [90].

Naz et al. developed FLX–dextran nanoparticles conjugates for better efficacy of FLX. Dextran was oxidized by sodium iodate to form the corresponding aldehyde, which was readily reacted with FLX, forming a Schiff base [91,92]. FLX–dextran nanoparticles were stable at physiological blood circulation and normal tissues with remarkably higher release in an acidic environment, i.e., pH 5, which resulted in high specificity toward cancer cells and reduced systemic undesirable effects. Indeed, only FLX showed higher toxicity to normal mouse embryo fibroblast cells 3T3 than FLX–dextran nanoparticles. However, the latter showed a similar anticancer effect on HeLa cells by completely inhibiting cellular growth at 30 µM concentration [91].

### 3.6. FLX in NSCLC

NSCLC represents 85% of lung cancer cases, and it is characterized by an invasive and metastatic nature; the acquired resistance to receptor tyrosine kinase inhibitors is a major therapeutic obstacle [93]. A research group has extensively studied the effect of FLX on NSCLC cells CL1-5-F4. FLX reduced their viability via induction of apoptosis in a dose-dependent manner with IC_50_ 40 µM, as shown by MTT assay. A Western blot analysis revealed that FLX significantly reduced the level of DNA repair-associated proteins, including levels of mediator of DNA damage checkpoint 1 (MDC1), O^6^-methylguanine DNA methyltransferase MGMT, and 14-3-3. Reporter gene assay showed that FLX remarkably inhibited NF-ĸB activation in CL1-5-F4 cells at the IC_50_ concentration of FLX. The related level of metastasis-associated proteins MMP-2, MMP-9, uPA, and VEGF was suppressed. Accordingly, CL1-5-F4 metastasis and invasion were attenuated [94].

In light of the above data, Hsua et al. benchmarked FLX in vivo using a CL1-5-F4-bearing mice model. FLX significantly suppressed tumor growth and size in the established model without general toxicity at 10 mg/kg FLX dose. The anticancer effect is ascribed to promoting the level of extrinsic and intrinsic apoptosis-associated proteins, including caspase-3, caspase-8, and caspase-9, and reduction of metastasis-associated protein VEGF, MMP-9, urokinase-type plasminogen activator (uPA), and Cyclin-D1. This validated the previous conclusion of FLX’s ability to attenuate NSCLC angiogenesis and invasion [94]. Additionally, FLX attenuated phosphorylation of NF-κB p65 on ser276, AKT, and ERK [80].

Another in vivo study by Yang et al. revealed that FLX (20 mg/kg) could suppress tumor growth as represented by weight and size in a xenograft mice model bearing subcutaneous transplanted lung cancer cells A549. While exposure to chronic unpredictable mild stress reduced serotonin and tryptophane concentrations and suppressed immunity, FLX treatment significantly reversed those effects. FLX promoted cellular immunity and elevated tryptophan/kynurenine ration. A biochemical assessment of kynurenine cascade-related genes tryptophan-2,3-dioxygenase (TDO), indoleamine-2,3-dioxygenase 1 (IDO1), and IDO2 showed that FLX (15 μmol/L) suppressed their upregulation due to chronic stress exposure [95].

### 3.7. FLX in Pancreatic Cancer

Pancreatic cancer is a relatively uncommon cancer that is characterized by a complex microenvironment. Its incidence is increasing, and it is expected to become the second leading cause of cancer-related death by 2030 [96]. As a hallmark of FLX safety to normal pancreatic cells, its administration did not induce acute pancreatitis compared to citalopram and other SSRIs [97]. A rough in vivo study on a subcutaneous xenograft model of human pancreatic cancer cell line SW1990 in mice revealed that FLX 10 mg/kg did not induce a significant reduction in tumor growth [98].

However, another impressive study by Schneider et al. showed a promising role of FLX in the management of pancreatic cancer via a serotonergic pathway. De facto, platelet-derived peripheral serotonin enhances the growth of murine pancreatic cancer cells Panc02. This is mediated by the enhanced expression of PD-L1 on mouse and human cancer cells in vitro via serotonylation reaction by covalently binding to glutamine amino acid, resulting in the activation of small G proteins. In turn, this impairs the accumulation of immune defense cells CD8^+^T and upregulates PD-L1 expression in the pancreatic cancer tumor microenvironment. Inhibition of serotonin cargo in platelets by FLX 20 mg/kg or TPH1 inhibitor telotristat reduced colon and pancreatic tumor growth in an established C57BL/6 mice model by increasing CD8^+^T accumulation within the tumor [99]. The authors concluded that combining FLX with anti-PD-1 therapy could be a potential way to treat solid tumors [99].

### 3.8. FLX in Lymphoma

Lymphomas are a heterogeneous group of malignancies that are attributed to the inadequate proliferation of lymphocytes at different maturation stages and account for around 5% of malignancies [100,101]. Human Burkitt lymphoma (BL) is an aggressive form of lymphoma due to the rapid proliferation rate, and it needs immediate intervention with strongly efficient chemotherapeutics [102]. FLX 50 µM exhibited quick apoptotic-mediated cytotoxicity against the chemo-sensitive BL cells MUTU-I, accompanied by an insignificant effect on normal blood cells. Independent of the serotonergic effect of FLX, it induced apoptosis via the caspase pathway, DNA cleavage, and PARP cleavage [103].

Later on, the same group explored the detailed mechanism using chemoresistant BL cell line DG-75 alongside MUTU-I. The former lacks Bax and Bak, making it difficult for any chemotherapy to induce apoptosis. FLX at 50 µM induced autophagy programmed cell death in DG-75 independently from the caspase pathway, DNA cleavage, and PARP cleavage. In DG-75 cells, Western blot showed upregulation of the autophagy marker Beclin 1, and the cytotoxic effect of FLX was reversed by cotreatment with the autophagy inhibitor 3-Methyladenine, contrary to MUTU-I cells. Extracellular Ca^2+^ influx was found to be crucial in FLX effect on DG-75 but not MUTU-I cells. Furthermore, the authors ruled out ROS involvement in BL cell death. Conclusively, in the chemo-sensitive cells MUTU-I, FLX elicits classic type I cell death apoptosis; nevertheless, it triggers type II cell death autophagy in the chemo-resistant cells DG-75 [104].

In EL4 lymphoma C57BL/6J mice model, FLX 15 mg/kg attenuated tumor growth accelerated by mice exposure to chronic stress conditions. Cell cycle regulators cyclins A2, D1, and D3 were elevated, whereas FLX treatment restored their mRNA expression levels to control values. Furthermore, FLX reduced invasiveness and metastasis to the liver and kidney. Once again, FLX reversed the effect of chronic stress by reducing MMP-2 and MMP-9 and increasing tissue inhibitors of metalloproteases TIMPs levels. Intriguingly, treatment with FLX promoted the antitumor immune response in animals [105]. Finally, It is worth noting that one recent report showed that FLX induced autophagy and apoptosis in lymphocytic leukemia targeting Akt phosphorylation dose-dependently [106]. The key findings in this review were summarized in Table 1.

### 3.9. FLX in Multidrug Resistance (MDR)

MDR operated by extrusion pumps such as P-glycoprotein is one of the main reasons for chemotherapy failure [107,108,109]. One of the earliest studies found that FLX works as a highly effective chemo-sensitizer by elevating the cytotoxicity of the anticancer drugs doxorubicin, mitomycin C, vinblastine, and paclitaxel in drug-resistant cancer cells. FLX modulated MDR in vitro and in vivo by slowing down the efflux rate of the titled chemotherapeutics [110].

Recent reports showcased that MDR is caused by the overexpression of transporters on the cancer cell membrane that expel chemotherapy out of the tumor cells. These pumps are members of the ATP-Binding Cassette (ABC) transporters superfamily [111]. There are seven subfamilies belonging to ABC viz., ABCA—ABCG, where ABCC includes 13 proteins, 9 of which are indicated as MDR proteins (MRPs). Recently, ABCC1/MRP1 and ABCC10/MRP7 were identified as the main drug transporters in MDR, and their inhibition is a potential tool for reversing MDR [112,113].

An interesting new study by Kanner et al. assessed three SSRIs, including FLX, for the binding and inhibition of MRP1 and MRP7 in silico. They found that FLX is well adopted in the binding sites of MRP1 and MRP7, with a slightly higher affinity toward MRP7. Experimentally, they found that FLX reversed the MDR resistance of MRP1 overexpressing human epidermoid carcinoma cell line KB/CV60 to vincristine and doxorubicin. In parallel, the same sensitizing effect was observed for MRP7-overexpressing human ovarian adenocarcinoma cell line SKOV3/MRP7, which is resistant to paclitaxel [114]. Conclusively, FLX could be an efficient chemo-sensitizer for overcoming MDR.

### 3.10. Potential Anticancer Mechanisms of FLX

Although FLX showed a vast array of anticancer activities, so far, its serotonin-independent molecular mechanism is yet to be elucidated. This provoked us to retrieve potential targets of FLX using the Swiss Target Prediction tool [51]. Other than CNS targets, FLX showed a high possibility of targeting four different kinases, namely serine/threonine-protein kinase PIM1, tyrosine-protein kinase receptor FLT3, glycogen synthase kinase-3 beta (GSK-3β), and serine/threonine-protein kinase Aurora-A. The titled kinases are valid targets in cancer therapy [115,116,117,118].

**Figure 3:**
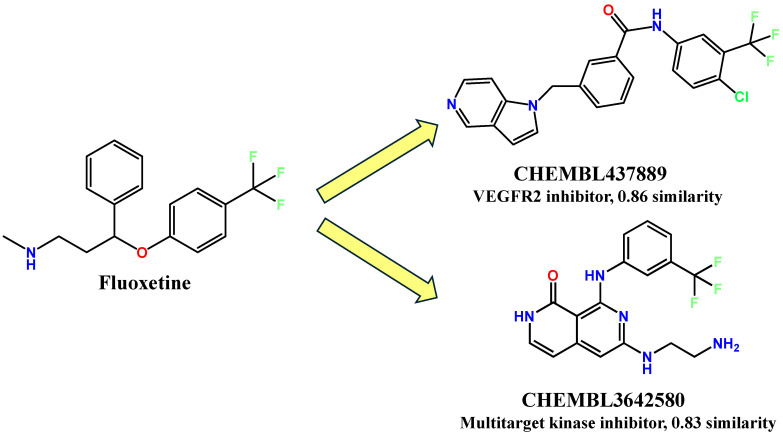
Similarity of fluoxetine (FLX) to a VEGFR2 inhibitor, CHEMBL437889; and a multitarget kinase inhibitor, CHEMBL3642580.

Indeed, FLX exhibited high 2D/3D similarities to inhibitors of those kinases, as predicted by SwissSimilarity [119]. Additionally, FLX showed structure similarity to vascular endothelial growth factor receptor 1 (VEGFR1) and vascular endothelial growth factor receptor 2 (VEGFR2) inhibitors, which are implicated in cancer angiogenesis and metastasis [120]. Other than kinases, FLX is predicted to inhibit the inhibitor of apoptosis protein 3 (IOA3) as well. Herein, we showcase an example of a reported molecule, CHEMBL437889, with a 0.86 3D similarity to FLX. According to SciFinder databases, CHEMBL437889 was reported to inhibit VEGFR1 and VEGFR2 with respective IC_50_ 0.4 µM and 0.3 µM [121,122]. In addition, a notable compound, CHEMBL3642580 [123], is a multitarget anticancer kinase inhibitor with a 3D similarity value of 0.83 to FLX (Figure 3). Conclusively, FLX has a high considerable similarity to oncolytic compounds, and its pharmacophores can be clues for medicinal chemists to be repurposed for cancer therapy.

## 4. Discussion and Future Perspectives and Challenges

This review of the literature showed that FLX has versatile oncolytic and chemo-sensitization effects with different mechanisms of action. In brain tumors, FLX showed activity against GBM and neuroblastoma. The effect of FLX against GBM was shown either as a monotherapy or as a sensitizer for another chemotherapy or radiotherapy. Seemingly, FLX sensitized GBM cells to TMZ via the CHOP and MGMT pathways. The coadministration of TMZ/FLX enhanced life expectancy in GBM patients. FLX showed pronounced chemo-sensitization in luminal breast cancer and TNBC. In doxorubicin-resistant MCF-7 cells, FLX simultaneously upregulated bcl-2 and p53. FLX enhances the cytotoxic effect of RAL by lowering VEGF, M-CSF, and MMP-9. In the same context, FLX proved to have a synergistic effect when combined with PXL and honeybee venom. Notably, the FLX effect extends to different TNBC phenotypes, such as SUM149PT, MDA-MB-231, and MDA-MB-436.

FLX showed in vitro and in vivo apoptotic effects on HEP3B HCC cells via increasing ROS generation and the suppression of related tyrosine kinases and metalloproteinases. HEX-loaded FLX was successfully used against HepG2 HCC cells. As per colorectal cancer, standalone FLX had an IC_50_ value of 6.12 µM against HT-29 cells with a significant synergetic effect with honeybee venom. Furthermore, FLX solely induced apoptosis in HCT116 cells via the p53-independent mechanism, whereas its combination with cisplatin or metformin/Efavirenz was more effective.

FLX reduced the viability of cervical cancer HeLa cells by increasing the cytosolic Ca^2+^ concentration and enhanced their sensitivity to cisplatin in vitro and in vivo by upregulating apoptotic proteins and downregulating MDR proteins. Using FLX–dextran nanoparticles conjugates improved its selectivity toward cancer cells over normal cells. In addition, FLX induced apoptosis and reduced invasiveness and angiogenesis in NSCLC cells CL1-5-F4 in a dose-dependent manner both in vitro and in vivo by upregulating different caspases and suppressing VEGF, MMP-9, urokinase-uPA, and Cyclin-D1. NSCLC A549 cells were sensitive to FLX treatment in vivo by promoting cellular immunity and suppressing TDO, IDO1, and IDO2 genes.

Surprisingly, a serotonin-dependent pathway was involved in the anticancer effect of FLX on murine pancreatic cancer cells Panc02. A serotonylation reaction triggers PD-L1 overexpression and increases CD8^+^T-cell accumulation in the cancer microenvironment, implying that FLX might be useful in combination with anti-PD-1 therapy. In another instance, FXR reduced the viability of both chemo-sensitive and chemo-resistant BL cells MUTU-I and DG-75, respectively. In the latter cells, FXR induced autophagy and extracellular Ca^2+^ influx without the induction of ROS or apoptosis.

Being an existing drug with an established pharmacokinetic and safety profile, FLX is a possible candidate for drug repurposing. However, most of its oncolytic properties are non-serotonin dependent and require high doses. The majority of in vivo studies used 10 mg/kg/day, which highly exceeds its recommended therapeutic dose, which is 20 mg and up to 80 mg per day. High doses of FLX can lead to severe and sometimes fatal serotonin syndrome [124]. Therefore, there is a long road ahead regarding FLX repurposing for oncolytic purposes. This comprises the elucidation of its molecular mechanism(s) and improving its potency to be more effective at lower doses.

## 5. Conclusions

Drug repurposing represents an indispensable tool for modern drug discovery that paves the way for a short-term and cost-effective bench-to-bedside drug transition. Several SSRIs have been benchmarked against a multitude of cancer types. FLX is distinguished by its highly favorable safety profile; compared to other SSRIs, it is unlikely to cause pancreatitis. Among different antidepressants, only FLX enhanced the overall survival of patients receiving antidepressant/PD-1/L1 immunotherapy combination compared to PD-1/L1 alone. Of note, FLX is characterized by its high repurposing potential, especially against GBM and neuroblastoma cells, which it can access readily due to its rapid BBB penetration ability. FLX exhibited cytotoxic effects against cancers from different origins, including breast, liver, colon, cervix, lung, pancreas, and lymph system cancers. We showed that FLX/TMZ combination action significantly surpasses monotherapy against GBM on both in vitro and in vivo levels. Similarly, the FLX/RAL combination outperforms monotherapy for the treatment of DMBA-induced breast cancer. Additionally, a combination of cisplatin with fluoxetine could be a judicious choice for the treatment of cervical cancer with a better outcome than cisplatin alone.

In this review, we further explained that FLX is a promising chemo-sensitizer that can circumvent the MDR of chemotherapy. We also discussed pharmaceutical preparation that loaded fluoxetine on carriers to improve its delivery and selectivity toward cancer cells that have a characteristic acidic environment. During the writing of this article, a new study was released showcasing the ability of FLX to inhibit the cell proliferation, invasion, metastasis, and angiogenesis of osteosarcoma cells by suppressing the phosphorylation of signal transducer and activator of transcription 3 (STAT3) [125]. Taken together, FLX repurposing could be a potential avenue for the cotreatment of cancer patients.

## Figures and Tables

**Table 1:** Summary of fluoxetine effect on cancer cells.

Cancer Origin	Cells	Conditions	Main Findings
Brain	U87	In vitro (25 μM) and in vivo (10 mg/kg)	Apoptosis by increasing the intracellular Ca^2+^ concentration via binding to AMPAR [53]
	C6, U373 and U251	In vitro	Respective IC50 14.7, 21.8, 48.5, and 22.9 μM. Apoptosis can be promoted by enhancing caspase-3 and CHOP concentrations and ERS [58]
	C6	In vitro, combined with TMZ	Synergistic antiproliferative effect mediated by CHOP pathway [58]
	U-138 MG	In vitro (10 μM) and in vivo, combined with TMZ	Synergistic antiproliferative effect by suppressing MGMT expression and disrupting NF-kB/p65 signaling [59]
	U87 RPE1, IMR90, and U87EGFRvIII	In vitro and in vivo (10, 15, and 16.4 mg/kg), alone or combined with TMZ	FLX reduced cell viability by suppressing SMPD1 and EGFRvIII dose-dependently. The combination is synergistic [55]
	Kelly cells	In vitro (10 μM), in vivo (20 mg/kg)	Inhibited CKS1, increased p27Kip1 expression, and triggered neuroblastoma cell death [60]
	U-87 MG	In vitro (10 μM), combined with radiotherapy	Radiosensitizer on cancer cells and radioprotective on normal cells HFFF2 [61]
Breast	MCF-7	In vitro	IC_50_ 7.78 µM [70]
	MCF-7	In vitro (10 µM) combined with PXL, doxorubicin, or honeybee venom,	Synergistic reduction of cell viability [70,71,72]
	MCF-7/ADM	In vitro (5 μg/mL) and combined with ADM	Significant enhancement of ADM cytotoxic effect with IC_50_ change from 13.62 µm to 2.7 µm. Effective reversal of ADM resistance and synergistic apoptosis via upregulation of p53 and downregulation of (Bcl-2) [65]
	(DMBA)-induced breast cancer	In vivo (30 mg/kg), combined with RAL	Synergistic antitumor effect and reduced concentration of TNF-α, IL-6, and TGF-β [68]
	SUM149PT	In vitro	Induced cytotoxicity (IC_50_ μM 7.9) and apoptosis and enhanced ER stress and autophagy with less effect on normal MCF-10A cells [74]
	MDA-MB-231 and MDA-MB-436	In vitro (1 μmol/L FLX)	Induced apoptotic and autophagic cell death via caspases, mTOR, and AMPK pathways [75]
Liver	Hep3B	In vitro (10, 30, and 100 µM)	Induced apoptosis, enhanced ROS formation, suppression of pERK1/2, and increased c-JNK dose-dependently [79]
	Hep3B	In vivo (10 mg/kg)	Antitumor effect by promoting caspases and suppressing VEGF, MMP-9, and NF-kB p65 [80]
	SK-Hep1	In vitro (30 μM)	Induction of apoptosis and reduced invasiveness through ERK/NF-κB suppression [81]
	HepG2	In vitro, FLX-loaded hexosome	Prolonged release with better efficacy than FLX [82]
Colon	HT-29	In vitro	IC_50_ 6.12 µM [70]
	HT-29	In vitro (10 µM), combined with honeybee venom	Synergistic antiproliferative effect [72]
	HCT116	In vitro (20 µM), combined with cisplatin or carboplatin	Synergistic antiproliferative effect by calmodulin inhibition [85]
	HCT116	In vitro (0.9 μM) and in vivo (2.67 mg/kg), combined with metformin/Efavirenz	Synergism, enhanced apoptosis, and autophagy with massive increase in ROS levels [86]
	p53 gene-depleted HCT116^−^/^−^	In vitro	IC_50_ 4.73 µM, cell-cycle arrest [87]
Cervix	HeLa cells	In vitro (50 μM)	Reduce viability by increasing cytosolic Ca^2+^ concentration [89]
	HeLa cells	In vitro (16 µM) and in vivo (3 mg/kg), combined with cisplatin (16 µM)	Synergism is achieved by upregulating apoptotic proteins and downregulating MDR proteins [90]
	HeLa cells	FLX–dextran nanoparticles	Similar cytotoxic effect of 30 μM FLX HeLa with less toxicity to normal cells 3T3 [91]
Lung	CL1-5-F4	In vitro	IC_50_ 40 µM by suppression of MMP-2, MMP-9, uPA, and VEGF [94]
	CL1-5-F4	In vivo (10 mg/kg)	Suppression of tumor growth and impeding angiogenesis and invasion [80]
	A549	In vivo (20 mg/kg)	Suppressed stress-induced tumor growth and kynurenine pathway and enhanced cellular immunity [95]
Pancreas	SW1990	In vivo (10 mg/kg)	Insignificant tumor growth suppression [98]
	Panc02	In vivo (20 mg/kg)	Suppression of tumor growth by upregulation of PD-L1 expression [99]
Lymph	MUTU-I	In vitro (50 μM)	Apoptosis via caspase pathway, DNA cleavage, and PARP cleavage [103]
	DG-75	In vitro (50 μM)	Autophagy via increasing Beclin-1 and extracellular Ca^2+^ influx [104]
	EL4	In vivo (15 mg/kg)	Attenuated tumor growth, invasiveness, and metastasis targeting cyclins, MMP-2 and MMP-9, and TIMPs [105]

## Data Availability

Not applicable.
